# Letter to the editor regarding Latent class analysis to predict intensive care outcomes in Acute Respiratory Distress Syndrome: a proposal of two pulmonary phenotypes

**DOI:** 10.1186/s13054-021-03604-7

**Published:** 2021-06-07

**Authors:** Karlijn J. P. van Wessem, Luke P. H. Leenen

**Affiliations:** Department of Trauma Surgery, University Medical Center Utrecht, Heidelberglaan 100, 3584 CX Utrecht, The Netherlands

**To the editor**,

With great interest we read the manuscript Latent class analysis to predict intensive care outcomes in Acute Respiratory Distress Syndrome: a proposal of two pulmonary phenotypes by Wendel Garcia et al. [1].

The authors have developed a proposal of two ARDS phenotypes based on a statistical method analyzing respiratory mechanics, gas-exchange and CT-derived gas-and tissue volume: Patients with *non-recruitable* phenotype 1 present with a lower respiratory system elastance, dead space and total lung tissue, as well as a higher PaO2/FiO2 ratio, a more physiological pH and a less inhomogeneous lung with a lower proportion of potentially recruitable lung than *recruitable* phenotype 2. Results showed that ICU mortality rate was higher in the *recruitable* than the *non-recruitable* phenotype even when corrected for PaO2/FiO2 ratio. The authors compare their newly developed phenotypes to other previously developed phenotypes (hypo-and hyper-inflammatory phenotypes) and they state that, although there was no direct correlation between the different phenotypes, there were some associations between high recruitability, and pulmonary inhomogeneity and the presence of increased pulmonary inflammatory biomarkers.

We acknowledge the authors for their effort in trying to develop personalized mechanical ventilation for ARDS, however we feel that they have artificially developed phenotypes based on a statistical method rather than on the pathophysiology of ARDS.

ARDS is characterized by an increased permeability of the alveolar-capillary barrier resulting in lung edema with protein-rich fluid causing an impairment of arterial oxygenation. Lung edema, endothelial and epithelial injury are accompanied by an influx of activated neutrophils into the interstitium and broncho-alveolar space [2, 3]. This increased permeability of the alveolar-capillary barrier combined with fluid filtration rates exceeding the capacity of the lung lymphatics for fluid removal leads to alveolar flooding, a major cause of hypoxemia in ARDS. Flooded lung units contribute to ventilation–perfusion mismatch and intrapulmonary shunt, all influencing lung recruitability.

The proposed phenotypes are artificially created phenotypes based on the same pathophysiological process. Mortality difference between both phenotypes is likely caused by the host’s reaction on the underlying inflammatory response. Since classification should serve clarification and guide therapy we see no additional value in another phenotype as it does not change our approach to the problem. Mechanical ventilation should already be personalized since it is depending on many different patient parameters. It would be far more promising to examine phenotypes based on the inflammatory response, since treatment based on the pathophysiology of ARDS will be likely the way to go [4].

## Data Availability

Not applicable.
