# Supplementary material for: Letter to the editor regarding Latent class analysis to predict intensive care outcomes in Acute Respiratory Distress Syndrome: a proposal of two pulmonary phenotypes
Source: Crit Care. 2021 Jun 7;25:195. doi: 10.1186/s13054-021-03604-7 (PMC8183037; doi:10.1186/s13054-021-03604-7)
Supplement: Related texts

# Reply: Response to: Letter to the editor regarding Latent class analysis to predict intensive care outcomes in Acute Respiratory Distress Syndrome: a proposal of two pulmonary phenotypes

**Authors:** Pedro D. Wendel Garcia, Alessio Caccioppola, Silvia Coppola, Tommaso Pozzi, Arianna Ciabattoni, Stefano Cenci, Davide Chiumello

We thank Dr. van Wessem and Dr. Leenen for their interest in our manuscript [1] and for their comments in response to it.

Since its coining in 1967, insights into the acute respiratory distress syndrome (ARDS) have evolved concurrent to the realization that the pathophysiological pathway, as described by Drs. van Wessem and Leenen, is an oversimplification of the heterogeneity governing ARDS. Neither do all patients with ARDS present histopathological signs of pneumonia with a high influx of activated neutrophils, as suggested by Drs. van Wessem and Leenen, nor do all lungs present diffuse alveolar damage (DAD) [5]. Considering this pathophysiological heterogeneity and the definition of a phenotype as *a clinical entity defined by observable characteristics without any mechanistic pathophysiological implication*, the claim by Drs. van Wessem and Leenen, that we “have artificially developed phenotypes based on a statistical method rather than on the pathophysiology of ARDS”, is rather surprising.

Drs. van Wessem and Leenen argue that the “mortality difference between both phenotypes is likely caused by the host’s reaction on the underlying inflammatory response “. Indeed the leading cause for mortality in ARDS, and consistently in the *hyper-inflammatory* phenotype, is severe sepsis and shock [5]. However, in patients presenting with DAD, concurrent to our *recruitable* phenotype, the cause of mortality is therapy refractory hypoxemia [6]. Notably, DAD presenting patients do not stand out due to their systemic inflammation or multiorgan dysfunction, but by a low PaO_2_/ FiO_2_ ratio and compliance, defining characteristics of our *recruitable* phenotype [6]. In fact, the alveolar concentration of most inflammatory mediators significantly surpasses their plasma concentration in ARDS, indistinctively of the underlying inflammatory phenotype, suggesting that the “currently known [inflammatory] phenotypes do not reflect the alveolar host response” [7].

Drs. van Wessem and Leenen conclude that “mechanical ventilation should already be personalized since it is depending on many different patient parameters”. Nonetheless, exactly the complex interaction of viscoelastic properties and host response, broadly correlating with the lungs morphological presentation, have impeded the successful personalization of mechanical ventilation [8]. To resolve this conundrum, identification of phenotypes reflecting the mechanical and morphological properties of the lung, like our proposed phenotypes, are necessary.

In conclusion, Drs. van Wessem and Leenen seem to neglect that ARDS is a syndrome of primarily pulmonary expression, and while we agree on the promise of inflammatory phenotypes to tailor systemic immunomodulating therapies, the cornerstone of ARDS management remains mechanical ventilation. Personalized research considering predictive enrichment through pulmonary focused phenotypes, such as our proposed *non-recruitable* and *recruitable* phenotypes, is of eminent importance to accomplish truly "individualized" mechanical ventilation.
